# Pretargeted immunoscintigraphy in patients with medullary thyroid carcinoma.

**DOI:** 10.1038/bjc.1996.443

**Published:** 1996-09

**Authors:** P. Magnani, G. Paganelli, C. Songini, A. Samuel, F. Sudati, A. G. Siccardi, F. Fazio

**Affiliations:** Institute H San Raffaele, University of Milan, Italy.

## Abstract

**Images:**


British Journal of Cancer (1996) 74, 825-831
© 1996 Stockton Press All rights reserved 0007-0920/96 $12.00

### Pretargeted immunoscintigraphy in patients with medullary thyroid carcinoma

P Magnani, G Paganelli*, C Songini, A Samuel, F Sudati, AG Siccardi and F Fazio

*INB-CNR, Institute H San Raffaele, University of Milan, Milan, Italy.*

**Summary** To evaluate the use of pretargeted immunoscintigraphy (ISG) in the diagnosis and follow-up of patients with medullary thyroid carcinoma (MTC), we studied 25 patients with histologically proven disease; ISG was repeated after surgery in two patients. The antibody, either an anticarcinoembryonic antigen (CEA) or an antichromogranin A (CgA) biotinylated monoclonal antibody (MAb) or a cocktail of the two biotinylated MAbs was first injected. After 24 h, avidin was administrated i.v., followed by $^{111}$In-labelled biotin 24 h later. Fifty-two lesions were visualised. Six primary tumours, diagnosed by increased calcitonin levels, were all correctly diagnosed; 47 recurrences, also suspected by blood tumour markers, were detected and confirmed by cytology or histology. In one case, single photon emission tomography allowed the detection of small lymph nodes with a diameter of 4–7 mm. These lesions, not judged neoplastic by ultrasound, were confirmed to be neoplastic by fine needle aspiration. Pretargeted ISG correctly localises primary tumours and recurrences in MTC patients, when the only marker of relapse is serum elevation of calcitonin. With this three-step pretargeting method, cocktails of potentially useful MAbs can be used, avoiding false-negative studies that may occur when CEA or CgA are not expressed.

**Keywords:** medullary thyroid carcinoma; monoclonal antibody; avidin–biotin

Medullary thyroid carcinoma (MTC) arises from calcitonin-secreting parafollicular cells in the thyroid. Both the sporadic and the familial form are treated by surgery (Chong *et al.*, 1975; Rossi *et al.*, 1980), as the efficacy of radiotherapy is limited (Samaan *et al.*, 1988) and chemotherapy is ineffective (Rougier *et al.*, 1983; Brunt and Wells, 1987). Early diagnosis of recurrence or metastasis and accurate localisation of recurrent disease are very important prerequisites for successful surgical excision.

Elevated serum calcitonin (Ct) is considered to be a marker for MTC. High serum levels of Ct and carcinoembryonic antigen (CEA) can often be found in patients with recurrent disease several years before it becomes clinically apparent, but, at present, no efficient and specific method is available for the localisation of recurrences.

Morphological imaging techniques (ultrasonography, US; computerised tomography, CT; magnetic resonance imaging, MRI) are routinely used to confirm and localise a biologically detected recurrence but are not always adequate because of topographic polymorphism and the small size of tumour recurrences at an early stage of development (Schwerk *et al.*, 1985; Frank *et al.*, 1987; Crow *et al.*, 1989).

Several methods based on tumour avidity of non-specific radiopharmaceuticals such as [$^{99m}$Tc](V)DMSA, $^{131/123}$I-MIBG and $^{201}$Tl have also been designed for this purpose, with variable results (Arnstein *et al.*, 1986; Hilditch *et al.*, 1986; Baulieu *et al.*, 1987; Clarke *et al.*, 1987; Hilditch, 1987; Clarke *et al.*, 1988; Hoefnagel *et al.*, 1988; Guerra *et al.*, 1989; Adams *et al.*, 1990; Charkes *et al.*, 1990; Udelsman *et al.*, 1993).

In the attempt to specifically localise tumour *in vivo*, MAbs directed to tumour-associated antigens and labelled with gamma-emitting isotopes can be used (Goldenberg *et al.*, 1980; Larson, 1985). The sensitivity of this technique can be enhanced by lowering the background with tumour pretargeting strategies (Hnatowich *et al.*, 1987; Goodwin *et al.*, 1988; Rowlinson *et al.*, 1988; Domogatsky, 1989; Le Doussal *et al.*, 1989; Kalofonos *et al.*, 1990; Peltier *et al.*, 1993). When tumour targeting is separated from injection of the radiolabel, injection of the radiotracer can be delayed to a time when circulating MAbs are removed from the blood and consequently background activity is low. Some of these strategies have the potential additional advantage of being able to target the tumour with different antibodies at the same time. One of these strategies, based on the avidin–biotin system, involves the injection of biotinylated MAbs (first step), followed by avidin administration (second step) to precipitate circulating biotinylated MAbs and, at the same time, to target the tumour cells for adequate homing in of the subsequently radiolabelled biotin (third step) (Paganelli *et al.*, 1991). In this case a cocktail of MAbs could be injected i.v. as first step.

Positive immunohistochemical stains for Ct and CEA are characteristic of MTC (De Lellis *et al.*, 1978; Talerman *et al.*, 1979; Pacini *et al.*, 1991). Recent immunohistochemical studies have established that MTC also stains positively for chromogranin A (CgA) (Schmid *et al.*, 1987), a glycoprotein stored and released by exocytosis together with the resident hormones of electron-dense core secretory granules in apudomas.

This paper reports the evaluation of pretargeted immunoscintigraphy (ISG) in the diagnosis of primary/metastatic and recurrent MTC. As antibodies, anti-CEA and anti-CgA (separately and as a cocktail) biotinylated MAbs were used in a three-step procedure.

### Materials and methods

### *Patients*

A continuous sequence of 25 patients attending endocrine and oncological clinics in various Italian cities and with a clinical diagnosis of MTC (ten female, 15 male; 18–68 years of age) were enrolled in the study, which was approved by the Scientific Institute HS Raffaele Ethics Committee. Before entering the study all patients gave written informed consent.

Six patients had primary tumour diagnosed by biochemical data and a family history of MTC. All six patients underwent surgery.

Eighteen patients had biochemical evidence of recurrence and one patient had US evidence of a thyroid remnant with

Correspondence: F Fazio, Department of Nuclear Medicine, Institute H San Raffaele, Via Olgettina 60, 20132 Milano, Italy
*Present address: Nuclear Medicine Unit, European Institute of Oncology
Received 19 October 1995; revised 18 March 1996; accepted 22 March 1996

Table I Clinical patient data

| Patients | Age (years)/ sex (M/F) | Familial (F)/ sporadic (S) | Syndrome | New case (NC)/ Recurrence (R) | Biochemical Ct b/s | data CEA[a] |
|---|---|---|---|---|---|---|
| 1 | 49/F | S | – | R | 20; 210 | Elevated |
| 2 | 43/M | S | – | R | 25; 230 | Elevated |
| 3 | 51/M | F | MEN2A | R | 15; 300 | Normal |
| 4 | 32/M | S | – | R | 50; 700 | Elevated |
| 5 | 19/F | S | MEN2B | R | 13; 300 | Elevated |
| 6 | 60/M | S | – | R | 647; 3000 | Elevated |
| 7 | 36/F | F | – | NC | 125; 2000 | Normal |
| 8 | 28/M | F | MEN2A | R | 1700; 3055 | Elevated |
| 9 | 29/M | S | – | NC | 24; 430 | Normal |
| 10 | 18/F | F | – | NC | 22; 205 | Normal |
| 11 | 40/M | F | – | NC | 13; 320 | Normal |
| 12 | 53/M | F | MEN2A | NC | 50; 400 | Elevated |
| 13 | 22/M | F | MEN2A | NC | 150; 3000 | Normal |
| 14 | 22/F | S | – | R | 314; 1700 | Elevated |
| 15 | 24/F | F | MEN2A | R | 513; 3300 | Normal |
| 16 | 29/M | F | MEN2A | R | 671; 3900 | Elevated |
| 17 | 46/F | S | – | R | 530; 2900 | NA |
| 18 | 26/F | F | MEN2A | R | 6; n.a. | NA |
| 19 | 54/M | S | – | R | 700; 2320 | Elevated |
| 20 | 46/M | F | – | R | 156; 1500 | Normal |
| 21 | 32/M | F | MEN2A | R | 20; 220 | Elevated |
| 22 | 22/F | F | MEN2A | R | 70; 400 | Elevated |
| 23 | 29/M | F | MEN2A | R | 1600; 2424 | Elevated |
| 24 | 34/M | F | – | R | 189; 1900 | Elevated |
| 25 | 68/F | S | – | | 160; 1800 | Elevated |

Normal value (IRMA): Ct < 10 pg $ml^{-1}$; CEA < 5 ng $ml^{-1}$. [a]Serum levels elevated or normal. NA, not available.

normal Ct serum levels following surgical treatment of their primary tumour. They underwent either fine needle aspiration (FNA) (five patients) or surgical intervention (14 patients) of all the lesions visualised by ISG.

Histopathological diagnosis (HIS) was always assessed on paraffin sections when surgery was available and on cytology when FNA had been performed.

ISG was repeated in two patients following surgery.

Clinical patient data are reported in Table I.

*Reagents*

MAb FO23C5 (IgG1) (Sorin Biomedica, Saluggia, Italy) reacts with the protein portion of the CEA molecule with an affinity constant ($K_a$) of $7 \times 10^8$ $M^{-1}$. It has already been described and extensively used in ISG (Buraggi *et al.*, 1987; Gasparrini *et al.*, 1988; Siccardi *et al.*, 1989).

MAb A11 (IgG1) is a monoclonal antibody directed against human CgA that does not react with CgB and CgC. It was obtained by immunisation of Balb/c mice with human phaeochromocytoma-derived chromaffin granules and screening of hybridoma supernatants by enzyme-linked immunoassay (ELISA) on crude chromogranin preparations. MAb A11 was characterised by two-dimensional immunoblotting and immunohistochemistry (Pelagi *et al.*, 1989) but $K_a$ is not yet defined.

MAbs were biotinylated by Società Prodotti Antibiotici (S.P.A., Milano, Italy) as previously described (Paganelli *et al.*, 1991). The degree of biotinylation was $5 \pm 1$ biotin per antibody determined spectrophotometrically, after protein digestion, as described (Hnatowich *et al.*, 1987). At this grade of biotinylation the retained immunoreactivity of the antibodies was more than 90%, as tested in a standard ELISA system (Paganelli *et al.*, 1991).

Pure hen egg avidin was obtained from S.P.A. DTPA-conjugated biotin was purchased from Sigma (St Louis, MO, USA).

*Radiolabelling*

DTPA-conjugated biotin was diluted in phosphate-buffered saline (PBS), pH 7.4, at a concentration of 2 $\mu g\ \mu l^{-1}$. The solution was sterilised by 0.22 $\mu m$ Millipore filtration. $^{111}InCl_3$ was diluted in citrate buffer (0.02 M; pH 6.5) to 740 kBq $\mu l^{-1}$. The reagents were then mixed and allowed to react at room temperature for 10 min. More than 98% of $^{111}In$ was bound to the conjugate, as shown by paper chromatography performed with Whatman no. 1 and bicarbonate buffer 0.05 M as liquid phase. The ability to bind avidin after labelling was verified by fast protein liquid chromatography (FPLC) (Pharmacia, Sweden), by mixing [$^{111}In$]biotin with an appropriate amount of avidin. No loss of reactivity was observed.

*Toxicity and immunogenicity*

All patients were closely observed for 2 h following administration of avidin. Blood samples (10 ml) were obtained in appropriate tubes just before the administration of avidin, then 10–15 days following the injection. All samples were sent out both for routine blood tests as well as to assess the human anti-mouse immunoglobulin (HAMA) and anti-avidin response (HAAR).

The induction of human anti-mouse immunoglobulin antibodies was investigated using an ELISA system (Seccamani *et al.*, 1989) in only 11 patients. In fact, our patients being a continuous sequence of patients attending endocrine and oncological clinics of various cities in Italy, they were not always willing to come back for blood tests. In these patients, avidin immunogenicity was studied on microwell plates coated with avidin or streptavidin separately. The plates were saturated for 1 h with PBS/3% bovine serum albumin (BSA). Human sera dilutions were added and incubated for 1 h at 37°C. After five washes, the binding of human anti-avidin antibodies was revealed with horseradish peroxidase-conjugated rabbit anti-human Ig antibodies (Dako) diluted 1:1000, for 45 min at 37°C. After six washes, the enzymatic reaction was developed with a chromogenic substrate (*o*-phenylendiamine; Sorin Biomedica, Saluggia, Italy) for 10 min and blocked by addition of 1 M sulphuric acid. The optical density reading was 492 nm.

*ISG study*

The three-step protocol used has been described previously (Paganelli *et al.*, 1991). Briefly: 1 mg of biotinylated MAb was injected i.v. over 2 min (first step). The biotinylated FO23C5 MAb was used in 16 ISG studies, the biotinylated A11 MAb in seven studies, whereas in four studies, a cocktail of biotinylated FO23C5 and A11 MAbs (0.5 mg+0.5 mg) was administered. After 24 h, 1 mg of unlabelled avidin was injected i.v. over 2 min, followed by an additional 9 mg 15 min later (second step). The aim of these two avidin administrations is to precipitate circulating biotinylated antibodies (1 mg) and subsequently to target the biotinylated MAbs bound on tumour cells (9 mg) (Schmid *et al.*, 1987; Buraggi *et al.*, 1987; O'Byrne *et al.*, 1992). [$^{111}$In]biotin (200 $\mu$g) (111–185 MBq) was injected i.v. 24 h after the administration of cold avidin in 3 ml of saline solution in a bolus injection (third step).

Within 1–3 h after the [$^{111}$In]biotin injection, a single photon emission tomography (SPET) study (64 x 64 pixel matrix, 64 projections over 360°) of the neck and planar spot views (64 x 64 pixel matrix, 150 Kcounts-view) of the neck, chest and abdomen were acquired. Indeed, as the label is a small molecule, background radioactivity levels are drastically reduced, and imaging can be performed shortly after injection of the radiolabel.

Images were obtained using a 40 cm circular field rotating gamma-camera (7500 Orbiter, Siemens), linked to a Microvax II computer (Siemens) and equipped with a high-energy collimator and by selecting two 15% energy windows centered over the 173 and 247 keV photopeaks of $^{111}$In.

Tomographic images were reconstructed with a filtered back projection algorithm and Hann filter (cut-off 0.5/pixel).

Planar and tomographic images were evaluated for the presence or absence of pathological tracer accumulation by two independent observers who are unaware of the clinical problem and the biochemical tumour marker levels. A semiquantitative visual analysis was carried out on planar and tomographic images by dividing the neck region into three sections (left laterocervical, median and right laterocervical). The ISG studies were visually scored; the score ranged from 0 to 2 (0=absent tracer uptake; 1=doubtful tracer uptake; 2=pathological tracer uptake). The statistical analysis was carried out on the scores of tracer uptake from the three sections considered. A kappa test (Fleiss, 1980) was used to test inter-observer agreement.

### Results

No toxicity was observed. Out of the 11 patients tested, one developed a weak antibody response against mouse immunoglobulins whereas six patients demonstrated anti-avidin antibodies 10–15 days after injection.

The kappa test demonstrated a strong agreement between the two observers ($\kappa>0.75$) both for planar and for tomographic images.

ISG results were classified as true positive (TP), true negative (TN), false positive (FP) and false negative (FN) according to histological diagnosis.

ISG results, final diagnoses and classifications are reported in Table II.

Figure 1 reports the overall distribution of activity in a typical patient. Activity biodistribution in the three-step pretargeting method using anti-CEA MAbs was compared with that obtained in the conventional ISG using the same antibody as Paganelli *et al.* (1991). No liver uptake and a negligible bone marrow uptake were demonstrated.

Fifty-two lesions were visualised by SPET and all verified by histology or cytology. Only three of these 52 lesions were visualised on planar spot views (patients 2, 16 and 23).

Fifty lesions were TP and two FP. In these two cases (patients 20, 21), metastatic lymph nodes visualised by ISG were not found at surgery. The smallest lesion detected by SPET was a right laterocervical lymph node of 4 mm in diameter. Two lymph nodes (patients 6, 21) identified as MTC metastases at histology were not visualised by ISG (two FN).

As regards patient 9 (TN, Table II), he had an abnormal Ct response to pentagastrin stimulation; ISG study did not show abnormal thyroid uptake of the tracer. The patient underwent surgery, and histology revealed a nodular hyperplasia as a colloid goiter.

**Table II** ISG results, final diagnoses and classifications

| *Patients* | *MAb (F)/(A)/(FA)* | *ISG sites of tracer uptake* | *Final diagnosis* | *HIS* | *Classification* |
|---|---|---|---|---|---|
| 1 | F | R | FNA | MTC | 1 TP |
| 2 | F | 2 left LC | FNA | MTC | 2 TP |
| 3 | F | 2 right LC | FNA | MTC | 2 TP |
| 4 | F | 2 right L:C | FNA | MTC | 2 TP |
| 5 | F | R | Histology | MTC | 1 TP |
| 6 | F | R + 1 left LC | Histology | MTC | 2 TP, 1 FN |
| 7 | F | Thyroid | Histology | MTC | 1 TP |
| 8 | F | 1 left + 1 right LC | Histology | MTC | 2 TP |
| 9 | F | Negative scan | Histology | NH | 1 TN |
| 10 | F | Thyroid | Histology | MTC | 1 TP |
| 11 | F | Thyroid | Histology | CCH | 1 TP |
| 12 | F | Thyroid + 3 right LC | Histology | MTC | 4 TP |
| 13 | F | Thyroid | Histology | MTC | 1 TP |
| 14 | F | R | Histology | MTC | 1 TP |
| 15 | A | R + 1 right LC | Histology | MTC | 2 TP |
| 16 | A | 2 right LC | Histology | MTC | 2 TP |
| 17 | A | R + 1 right LC | Histology | MTC | 2 TP |
| 18 | A | R + 2 left LC | Histology | MTC | 3 TP |
| 19 | A | R + 2 left, 3 right LC | Histology | MTC | 6 TP |
| 20 | A | R + 3 left LC | Histology | MTC | 3 TP, 1 FP |
| 21 | A | R + 1 right LC | FNA | MTC | 1 TP, 1 FP, 1 FN |
| 22 | FA | 2 left, 1 right LC | Histology | MTC | 3 TP |
| 23 | FA | R + 1 left, 1 right LC | Histology | MTC | 3 TP |
| 24 | FA | 2 right LC | Histology | MTC | 2 TP |
| 25 | FA | 2 right LC | Histology | MTC | 2 TP |
| Total | | 11R, 5 thyroid, 36 LC | | | 50 TP, 1 TN, 2 FP, 2 FN |

MAb, (F) FO23C5; (A) A11; (FA) FO23C5 + A11. NH, nodular hyperplasia; CCH, C-cell hyperplasia; R, remnant; LC, laterocervical lymph nodes.

The two follow-up ISG studies correctly identified the presence of malignant tissue.

Patient 7 had a primary MTC with elevated Ct serum levels and a first positive ISG study. The tumour was removed by surgery, and histology confirmed two MTC foci. Immunohistochemical staining was highly positive for CEA. Ct serum levels returned to normal post-operatively (basal and in response to pentagastrin stimulation) and post-operative ISG showed no cervical accumulation.

Patient 4, who had biochemical evidence of recurrence following surgical treatment of his primary MTC, underwent a first ISG study that showed a pathological tracer uptake in the right region of the neck. US did not confirm a laterocervical involvement and FNA was inadequate. ISG was repeated about 4 months later and an abnormal tracer uptake was still evident in some right laterocervical lymph nodes. FNA was also repeated and cytology confirmed the presence of metastases of MTC.

Four patients who underwent anti-CEA ISG study had normal CEA serum level and a TP scan. In particular, patient 11 with C-cell hyperplasia had a positive ISG study. This patient, with a family history of MEN2A and elevated Ct serum levels in response to pentagastrin stimulation, underwent total thyroidectomy. Histology revealed diffuse C-cell hyperplasia with an immunohistochemical stain positive for CEA. On the other hand, patient 18 had normal Ct serum levels, but US revealed a thyroid remnant. ISG showed a strong uptake of the tracer not only in the residual thyroid but also in some left laterocervical lymph nodes (Figure 2). The patient underwent surgery and histology confirmed the presence of metastases of medullary thyroid carcinoma.

Figure 3 illustrates a case of cervical involvement (patient 3) detected by ISG. Immunoscintigraphy revealed a clear uptake of the tracer in the right region of the neck. Two small lymph nodes of 4 mm and $11 \times 7$ mm in diameter, not detected as neoplastic by US, have been confirmed to be metastases from MTC by FNA. Moreover, this patient had a suspicious-looking solid lesion, 20 mm in diameter in the liver, revealed by US and CT. ISG showed no liver uptake. Histological control revealed a benign liver lesion (angioma).

Figures 4–6 show examples of planar and SPET images from patient 15, 18 and 25 respectively.

### Discussion

Medullary thyroid carcinoma is an uncommon tumour that frequently metastasises to cervical and mediastinal lymph nodes, bone and lung (Brunt and Wells, 1987). Ct and CEA are very sensitive indicators of MTC and are currently used as serum markers for relapse or distant metastases after thyroidectomy. Localisation of the tumour may be a more

**Figure 1** Whole-body biodistribution obtained 2 h after the injection of radioactivity. Most of the activity is already excreted by the kidneys (k) with high levels in the bladder (B). Please note the absence of activity in the liver and skeleton. Some biotin is also excreted in the mammary glands (arrow). R, right.

**Figure 2** ISG SPET study of patient 18, performed 2 h after radioactivity injection. Transaxial sections go from bottom to top. A strong uptake of the tracer in the thyroid remnant is clearly evident in front of the trachea (T), which appears as a cold area in the transaxial sections. The study also revealed a pathological uptake in some laterocervical lymph nodes (arrow). Histology confirmed the presence of metastases of medullary thyroid carcinoma. R, right.

**Figure 3** US study (**a**) of patient 3: two small lymph nodes, the larger of them $6 \times 4$ mm in diameter, are visible in the right region of the neck (arrow). Pretargeted ISG coronal sections (**b**), performed 2 h after $[^{111}In]$biotin injection. Coronal sections go from posterior to anterior view. A pathological uptake of the tracer is evident in the right cervical region (arrows) and corresponds to cervical lymph nodes. FNA was performed and cytology confirmed the presence of metastases from MTC. R, right.

complicated problem. US is useful in the post-surgical follow-up of MTC patients in order to evaluate cervical lymph nodes and relapse, but it is not specific (Schwerk *et al.*, 1985). MRI has similar indications and it can also be used in the evaluation of the mediastinum (Crow *et al.*, 1989). Various radiotracers have been proposed for this purpose. [$^{99m}$Tc](V)DMSA, $^{131/123}$I-MIBG and $^{201}$Tl show different sensitivities of lesion detection in different reports (Arnstein *et al.*, 1986; Hilditch *et al.*, 1986, 1987; Baulieu *et al.*, 1987; Clarke *et al.*, 1987, 1988; Hoefnagel *et al.*, 1988; Adams *et al.*, 1990; Guerra *et al.*, 1989; Charkes *et al.*, 1990; Udelsman *et al.*, 1993). These different sensitivities can be related to patient selection, limited number of patients studied, Ct blood levels, dimension of lesions studied or to methodological problems. For example, Clarke *et al.* (1987, 1988) reported a sensitivity of 74% in 19 patients using [$^{99m}$Tc](V)DMSA, Mojiminiyi *et al.* (1989) correctly diagnosed seven out of eight patients, Hilditch *et al.* could not reveal abnormal foci of tracer uptake in any of five MTC patients in a first work (Hilditch *et al.*, 1987), whereas in a subsequent work (Hilditch *et al.*, 1988) they correctly diagnosed three out of four MTC patients. These are certainly good results; however, they were obtained in too few patients.

**Figure 4** ISG planar image (A) and transaxial sections (B, C) from patient 18. No pathological tracer uptake is evident on planar spot view. A pathological tracer uptake is clearly evident in a left laterocervical lymph node (B, arrow) and in the thyroid remnant (C, arrow) on SPET sections. R, right.

**Figure 5** ISG planar image (A) and transaxial section (B) from patient 15. A pathological tracer uptake in the thyroid remnant (arrow) is visible only in the SPET section. R, right.

**Figure 6** ISG planar image (A) and coronal section (B) from patient 25. A pathological tracer uptake in two right laterocervical lymph nodes (arrows) is visible only in the SPET section. R, right.

*In vivo* tumour localisation can be obtained using radiolabelled monoclonal antibodies directed against tumour-associated antigens. Thus ISG is very specific, but a relatively low tumour to background ratio and a high background activity can affect its sensitivity. Theoretically ISG can target different MTC-associated antigens, such as Ct, CEA and CgA (De Lellis *et al.*, 1978; Talerman *et al.*, 1979; Schmid *et al.*, 1987; Pacini *et al.*, 1991). MTC often produces CEA, which is expressed both in the cytoplasm and on the cell membrane. On the contrary, Ct is expressed within the cytoplasm. In our pretargeting protocol, the most appropriate target antigen must be available on the cell surface. This happens because biotinylated MAb binds to the antigen and becomes, in its turn, the 'antigen' for the cold avidin and the radiotracer. Recently, a positive immunostaining for CgA in MTC has also been described: the staining for CgA correlates with the areas staining positively for calcitonin (Schmid *et al.*, 1987). CgA is released by neuroendocrine tumours without physiological neuroendocrine stimuli and is present in a significant amount in the intracellular matrix of such tumours.

Several studies have shown the capability of ISG performed with $^{131}$I or $^{111}$In-labelled anti-CEA MAb to visualise MTC recurrences (Berche *et al.*, 1982; Reiners *et al.*, 1986; Edington *et al.*, 1988; Manil *et al.*, 1989; Zanin *et al.*, 1990; O'Byrne *et al.*, 1992; Vuillez *et al.*, 1992). However, after injection of directly labelled MAb, image interpretation is sometimes difficult because of non-specific bone marrow, vascular and liver activity (Peltier *et al.*, 1993). In CEA-positive tumours, gliomas and lung cancers (Paganelli *et al.*, 1994*a*; Dosio *et al.*, 1994), and more recently in neuroendocrine tumours using an anti-CgA MAb (Colombo *et al.*, 1993), the avidin–biotin pretargeting method provides good results and offers several advantages over the administration of directly labelled MAbs. A fast label clearance and removal of circulating antibodies, with low background radioactivity levels, and the preservation of MAb immunoreactivity (Paganelli *et al.*, 1991) were demonstrated. In addition the second and third step of this three-step protocol can be common to all studies, irrespective of the specificity of the anti-tumour MAb, and potentially useful MAbs can be injected in sequence or even in combination, as we did, as the first step. In this way, radiolabelled biotin can serve as a single carrier of diagnostic or therapeutic radionuclides in patients receiving cocktails of antibodies.

In the present study, four patients underwent ISG using, for the first step, a cocktail of MAbs that recognises different tumour-associated antigens (CEA and CgA) (Fazio and Paganelli, 1993; Matzku *et al.*, 1989). As the activity delivered to the tumour is low, the use of a cocktail would enhance the possibility of targeting more tumour cells by using different tumour antigens as targets. Thus, using a cocktail of antibodies we could either avoid false-negative studies that may occur when one of the two antigens is not expressed, or provide an amplification of the signal from the tumour, given that more avidin can bind biotinylated MAbs that recognised the two antigens expressed in different tumour cells. Of the 55 histologically confirmed ISG lesions, 50 turned out to be TP, two TN, two FP and two FN. One patient (no. 7), who underwent ISG 2 months after surgery for primary MTC, did not show tracer uptake. Ct serum levels, both basal and after pentagastrin stimulation, and neck US were normal in a 6 month period of follow-up. Thus the patient can also be considered TN.

No relationship between CEA serum levels and ISG sensitivity was found. Four patients who underwent anti-CEA ISG studies had normal CEA serum levels and TP scans. Thus, ISG using anti-CEA MAb is effective even when Ct alone is elevated.

All primary tumours were visualised. In addition, a patient with C-cell hyperplasia, considered a preneoplastic condition, was correctly diagnosed, and in a patient with normal Ct serum levels a pathological uptake of the tracer was demonstrated by ISG and subsequently confirmed by histology.

Fifty out of 52 tumour recurrences (96%) were imaged and confirmed by cytology or histology.

Tomographic ISG study was crucial for detecting small lymph nodes, with a diameter of 4–7 mm, that were not recognised as neoplastic by US. Moreover the presence of blood activity, although low with respect to directly labelled MAbs, could make it difficult to distinguish lymph node activity from vascular background (see Figures 4–6) on planar images, whereas using SPET the separation of tumour from background activity is feasible.

A patient whose US and CT scans revealed a liver lesion had a negative ISG liver scan, and histology subsequently revealed the benign nature of the lesion.

One potential disadvantage of this pretargeting protocol is the immunogenicity of avidin. The immunogenicity of biotinylated antibodies and avidin was tested in only 11 patients. However, data available from 60 other patients receiving, in similar protocols, biotinylated antibodies (i.e. anti-tenascin, anti-CEA and anti-CgA) and avidin, comply with the results of this study. In particular, none of the patients studied developed an important response to mouse immunoglobulin after the injection of 1 mg of biotinylated IgG and 22% developed HAAR after the injection of 5–6 mg of avidin (unpublished data).

In conclusion, pretargeted ISG with CEA and/or CgA MAb may be useful in MTC follow-up, when the only marker of relapse is serum elevation of Ct, in order to localise tumour recurrences, and in the early detection of preneoplastic conditions, such as C-cell hyperplasia.

With this method, radiolabelled biotin can serve as a carrier not only for diagnostic but also for therapeutic purposes. However, radioactivity delivered per gram of tumour is still below the optimal dose for radioimmunotherapy (Paganelli *et al.*, 1994*b*). A higher tumour radioactivity could be obtained by using more biotinylated MAbs in the first step and streptavidin in the second step, as this results in a better avidination of the tumour. As avidin blood clearance is very fast, the longer plasma $t_{1/2}$ of streptavidin can convey more streptavidin and so more radiolabelled biotin to the tumour. However, streptavidin may be more immunogenic than avidin when injected in humans (unpublished data). Methods to block this response are currently under evaluation and it is anticipated that advances in molecular biology and recombinant DNA technology will contribute significantly to circumventing this problem. By optimising the radioactivity channelled onto the tumour and by labelling biotin with $\beta$-emitting isotopes, such as $^{90}$Y and $^{188}$Re, antibody-guided therapy of small MTC recurrences may be feasible.

**Acknowledgement**
The work was supported in part by grant of the Consiglio Nazionale delle Ricerche, Finalised Project ACRO.

### References

ADAMS BK, FATAAR A, BYRNE MJ, LEVIT NS AND MATLEY PJ. (1990). Pentavalent technetium-99m (V) DMSA uptake in a patient with Sipple's syndrome. *J. Nucl. Med.*, **31**, 106–108.

ARNSTEIN NB, JUNI JE, SISSON JC, LLOYD RV AND THOMPSON NW. (1986). Recurrent medullary carcinoma of the thyroid demonstrated by thallium-201 scintigraphy. *J. Nucl. Med.*, **27**, 1564–1568.

BAULIEU JL, GIULLOTEAU D, DELISLE MJ, PERDRISOT R, GARDET P, DELEPINE N, BAULIEU F, DUPONT JL, TALBOT JN, COUTRIS G AND CALMETTES C. (1987). Radioiodinated meta-iodobenzylguanidine uptake in medullary thyroid cancer. A French cooperative study. *Cancer*, **60**, 2189–2194.

BERCHE C, MACH JP AND LUMBROSO JD. (1982). Tomoscintigraphy for detecting gastrointestinal and medullary thyroid cancer: first clinical results using radiolabelled monoclonal antibodies against carcinoembryonic antigen. *Br. Med. J.*, **285**, 1447–1451.

BRUNT LM AND WELLS SA. (1987). Advances in the diagnosis and treatment of medullary thyroid carcinoma. *Endocrinol. Surg.*, **67**, 263–279.

BURAGGI GL, CALLEGARO L, TURRIN A, GENNARI L, BOMBARDIERI E, MARIANI G, DELEIDE G, DOVIS M, GASPARINI M, DOCI R, REGALIA E, SEREGNI E AND SCASSELLATI GA. (1987). Immunoscintigraphy of colorectal carcinoma with F(ab')$_2$ fragments of anti-CEA monoclonal antibody. *Cancer Detect. Prev.*, **10**, 335–345.

CLARKE SEM, LAZARUS C, MISTRY R AND MAISEY MN. (1987). The role of technetium-99m pentavalent DMSA in the management of patients with medullary carcinoma of the thyroid. *Br. J. Rad.*, **60**, 1089–1902.

CLARKE SEM, LAZARUS CR, WRAIGHT P, SAMPSON C AND MAISEY MN. (1988). Pentavalent (99mTc)DMSA, (131I)MIBG, and (99mTc)MDP: an evaluation of three imaging technique in patients with medullary carcinoma of the thyroid. *J. Nucl. Med.*, **29**, 33–38.

CHARKES ND, VITTI RA AND BROOKS K. (1990). Thallium-201 SPECT increases detectability of thyroid cancer metastases. *J. Nucl. Med.*, **31**, 147–153.

CHONG GC, BEAHRS OH, SIZEMORE GW AND WODNER LH. (1975). Medullary carcinoma of the thyroid gland. *Cancer*, **35**, 695–704.

COLOMBO P, PAGANELLI G, MAGNANI P, SONGINI C, FAZIO F AND FAGLIA G. (1993). Immunoscintigraphy with anti-chromogranin A antibodies in patients with endocrine/neuroendocrine tumors. *J. Endocrinol. Invest.*, **16**, 841–843.

CROW JP, AZAR-KIA B AND PRINZ RA. (1989). Recurrent occult medullary thyroid carcinoma detected by MR imaging. *Am. J. Roentgenol.*, **152**, 1255–1256.

DE LELLIS RA, RULE AH, SPILER I, NATHANSON L, TASHJIAN AH AND WOLFE HJ. (1978). Calcitonin and carcinoembryonic antigen as tumour markers in medullary thyroid carcinomas. *Am. J. Clin. Pathol.*, **70**, 587–594.

DOMOGATSKY SP. (1989). Rapid blood clearance of biotinylated IgG after infusion of avidin. *J. Nucl. Med.*, **30**, 66–69.

DOSIO F, MAGNANI P, PAGANELLI G, SAMUEL A, CHIESA G AND FAZIO F. (1994). Three-step tumour pretargeting in lung cancer. *J. Nucl. Biol. Med.*, **37**, 228–233.

EDINGTON HD, WATSON CG AND LEVINE G. (1988). Radioimmunoimaging of metastatic medullary carcinoma of the thyroid gland using an indium-111 labelled monoclonal antibody to CEA. *Surgery*, **104**, 1004–1010.

FAZIO F AND PAGANELLI G. (1993). Antibody-guided scintigraphy: targeting the 'magic bullet'. *Eur. J. Nucl. Med.*, **20**, 1138–1140.

FLEISS JL. (1980). The measurement of interrater agreement. In *Statistical Methods for Rates and Proportions*, pp. 212–236, J Wiley and Sons: New York.

FRANK K, RAUE F, LORENZ D, HEFARTH C AND ZIEGLER R. (1987). Importance of ultrasound examination for the follow-up of medullary thyroid carcinoma: comparison with other localization methods. *Henry Ford Hosp. Med. J.*, **35**, 122–123.

GASPARINI M, RIPAMONTI M, SEREGNI E, REGALIA E AND BURAGGI GL. (1988). Tumour imaging of colo-rectal carcinoma with an anti-CEA monoclonal antibody. *Int. J. Cancer*, (Suppl. 2), 81–84.

GOLDENBERG DM, KIM EE, DELAND FH, BENNET S AND PRIMUS FJ. (1980). Radioimmunodetection of cancer with radioactive antibodies to carcinoembryonic antigen. *Cancer Res.*, **40**, 2984–2992.

GOODWIN DA, MEARES CF, MCCALL MJ, MCTIGUE M AND CHAOVAPONG W. (1988). Pre-targeted immunoscintigraphy of murine tumours with indium-111-labelled bifunctional haptens. *J. Nucl. Med.*, **29**, 226–234.

GUERRA UP, PIZZOCARO C, TERZI A, GIUBBINI R, MAIRA G, PAGLIAINI R AND BESTAGNO M. (1989). New tracers for the imaging of the medullary thyroid carcinoma. *Nucl. Med. Commun.*, **10**, 285–295.

HILDITCH TE. (1987). Technetium-99m(V)-DMSA in the imaging of medullary thyroid carcinoma. *J. Nucl. Med.*, **28**, 253.

HILDITCH TE, CONNEL JMC, ELLIOT AT, MURRAY T AND REED NS. (1986). Poor results with technetium-99m(V) DMSA and iodine-131 MIBG in the imaging of medullary thyroid carcinoma. *J. Nucl. Med.*, **27**, 1150–1153.

HNATOWICH DJ, VIRZI F AND RUSCKOWSKI M. (1987). Investigation of avidin and biotin for imaging applications. *J. Nucl. Med.*, **28**, 1294–1302.

HOEFNAGEL CA, DELPRAT CC, ZANIN D AND VAN DER SCHOOT JB. (1988). New radionuclide tracers for the diagnosis and therapy of medullary thyroid carcinoma. *Clin. Nucl. Med.*, **13**, 159–165.

KALOFONOS HP, RUSCKOWSKI M, SIEBECKER DA, SIVOLAPENKO GB, SNOOK D, LAVENDER JP, EPENETOS AA AND HANTOWICH DJ. (1990). Imaging of tumour in patients with Indium-111-labelled biotin and streptavidin-conjugated antibodies: preliminary communications. *J. Nucl. Med.*, **31**, 1791–1796.

LARSON MS. (1985). Radiolabelled monoclonal anti-tumour antibodies in diagnosis and therapy. *J. Nucl. Med.*, **26**, 538–545.

LE DOUSSAL JM, MARTIN M, GAUTHEROT E, DELAAGE M AND BARBET J. (1989). In vitro and in vivo targeting of radiolabelled monovalent and divalent haptens with dual specificity monoclonal antibody conjugates: enhanced divalent hapten affinity for cell-bound antibody conjugate. *J. Nucl. Med.*, **30**, 1358–1366.

MANIL L, BOUDET F, MOTTE P, GARDET P, SACCAVINI JC, LUMBROSO JD, SCHLUMBERGER M, CAILLOU B, BAZIN JP, RICARD M, BELLET D, BOHUON C, TUBIANA M, DI PAOLA R AND PARMENTIER C. (1989). Positive anticalcitonin immunoscintigraphy in patients with medullary thyroid carcinoma. *Cancer Res.*, **49**, 5480–5485.

MATZKU S, KIRCHGESSNER H, SCHMID U, TEMPONI M AND FERRONE S. (1989). Melanoma targeting with a cocktail of monoclonal antibodies to distinct determinants of the human HMW-MAA. *J. Nucl. Med.*, **30**, 390–397.

MOJIMINIYI OA, UDELSMAN R, SHEPSTONE BJ, DUDLEY NE AND SOPER NDW. (1989). More on [$^{99m}$Tc](V)DMSA scintigraphy in patients with medullary carcinoma of the thyroid. *J. Nucl. Med.*, **30**, 1420.

O'BYRNE KJ, HAMILTON D, ROBINSON I, SWEENEY E, FREYNE PJ AND CULLEN MJ. (1992). Imaging of medullary carcinoma of the thyroid using $^{111}$In-labelled anti-CEA monoclonal antibody fragments. *Nucl. Med. Commun.*, **13**, 142–148.

PACINI F, BASOLO F AND ELISEI R. (1991). Medullary thyroid cancer: an immunohistochemical and tumoural study using six separate antigens. *Am. J. Clin. Pathol.*, **95**, 300–308.

PAGANELLI G, MAGNANI P, ZITO F, VILLA E, SUDATI F, LOPALCO L, ROSSETTI C, MALCOVATI M, CHIOLERIO F, SECCAMANI E, SICCARDI AG AND FAZIO F. (1991). Three-step monoclonal antibody tumour targeting in carcinoembryonic antigen-positive patients. *Cancer Res.*, **51**, 5960–5966.

PAGANELLI G, MAGNANI P, ZITO F, LUCIGNANI G, SUDATI F, TRUCI G, MOTTI E, TERRENI M, POLLO B, GIOVANELLI M, CANAL M, SCOTTI G, COMI G, KOCH P, MAECKE HR AND FAZIO F. (1994*a*). Pre-targeted immunodetection in glioma patients: tumour localization and single-photon emission tomography imaging of ($^{99m}$Tc)PnAo-biotin. *Eur. J. Nucl. Med.*, **21**, 314–321.

PAGANELLI G, SONGINI C, MAGNANI P, SAMUEL A, DI LEO C, SUDATI F, SIDOLI S, SICCARDI AG AND FAZIO F. (1994*b*). Pretargetted immunoscintigraphy with the avidin/biotin system: four years experience. *J. Nucl. Med.*, **35**, 55P.

PELAGI M, BISIANI C, GINI A AND BONARDI MA. (1989). Preparation and characterization of an anti-human chromogranin A and chromogranin B (secretogranin I) monoclonal antibodies. *Mol. Cell Probes*, **3**, 87–101.

PELTIER P, CURTET C, CHATAL JF, LE DOUSSAL JM, DANIEL G, AILLET G, GRUAZ-GUYON A, BARBET J AND DELAAGE M. (1993). Radioimmunodetection of medullary thyroid cancer using a bispecific anti-CEA/anti-Indium-DTPA antibody and an Indium-111-labelled DTPA dimer. *J. Nucl. Med.*, **34**, 1267–1273.

REINERS C, EILLES C, SPIEGEL W, BECKER W AND BORNER W. (1986). Immunoscintigraphy in medullary thyroid cancer using a $^{123}$-I or $^{111}$In-labelled monoclonal anti-CEA antibody fragment. *Med. Nucl.*, **25**, 227–231.

ROSSI RL, MEISSNER WA, WOD MS, SEDGWICK CE AND WERVER J. (1980). Nonfamilial medullary thyroid carcinoma. *Am. J. Surg.*, **139**, 554–560.

ROUGIER P, PARMENTIER C AND LAPLANCHE A. (1983). Medullary thyroid carcinoma: prognostic factors and treatment. *Int. J. Radiat. Oncol. Biol. Physiol.*, **9**, 161–169.

ROWLINSON G, RUSCKOWSKI M, GIONET M, SIEBECKER D, SNOOK D, EPENETOS AA AND HNATOWICH DJ. (1988). Animal tumour localization studies with streptavidin conjugated antibody and labelled biotin. *J. Nucl. Med.*, **29**, 762.

SAMAAN NA, SCHULTZ PA AND HICKEY RC. (1988). Medullary thyroid carcinoma: prognosis of familial versus sporadic disease and the role of radiotherapy. *J. Clin. Endocrinol. Metab.*, **67**, 8801–8805.

SCHMID KW, FISCHER-COLBRIE R, HAGN C, JASANI B, WILLIAMS ED AND WINKLER H. (1987). Chromogranin A and B and secretogranin II in medullary carcinomas of thyroid. *Am. J. Surg. Pathol.* **11**, 551–556.

SCHWERK WB, GRUN R AND WAHL R. (1985). Ultrasound diagnosis of C-cell carcinoma of the thyroid. *Cancer*, **55**, 624–630.

SECCAMANI E, TATTANELLI M, MARIANI M, SPRANZI E, SCASSELLATI GA AND SICCARDI AG. (1989). A simple qualitative determination of human antibodies to murine immunoglobulins (HAMA) in serum samples. *Nucl. Med. Biol.*, **2**, 167–170.

SICCARDI AG, BURAGGI GL, CALLEGARO L, CENTI-COLELLA A, DE FILIPPI PG, GALLI G, MARIANI G, MASI R, PALUMBO R, RIVA P, SALVATORE M, SCASSELLATI GA, SCHEIDHAUER K, TURCO GL, ZANIOL P, BENINI S, DELEIDE G, GASPARINI M, LASTORIA S, MANSI L, PAGANELLI G, SALVISCHIANI E, SEREGNI E, VIALE G AND NATALI PG. (1989). Immunoscintigraphy of adenocarcinomas by means of radiolabelled $F(ab')_2$ fragments of an anti-carcinoembryonic antigen monoclonal antibody: a multicenter study. *Cancer Res.*, **49**, 3095–3103.

TALERMAN A, LINDEMAN J, KIEVIT-TYSON PA AND DROGE-DROPPERT C. (1979). Demonstration of calcitonin and carcinoembryonic antigen (CEA) in medullary carcinoma of the thyroid (MTC) by immunoperoxidase technique. *Histopathology*, **3**, 503–510.

UDELSMAN R, BALL D, BAYLIN SB, WONG CY, OSTERMAN FA, AND SOSTRE S. (1993). Preoperative localization of occult medullary carcinoma of the thyroid gland with single-photon emission tomography dimercaptosuccinic acid. *Surgery*, **114**, 1083–1089.

VUILLEZ J, PELTIER P, CARAVEL JP, CHETANNEAU A, SACCAVINI JC AND CHATAL JF. (1992). Immunoscintigraphy using $^{111}$In-labelled $F(ab')_2$ fragments of anti-carcinoembryonic antigen monoclonal antibody for detecting recurrences of medullary thyroid carcinoma. *J. Clin. Endocrinol. Metab.*, **74**, 157–163.

ZANIN DEA, VAN DONGEN A, HOEFNAGEL CA AND BRUNING PF. (1990). Radioimmunoscintigraphy using iodine-131 anti-CEA monoclonal antibodies and thallium-201 scintigraphy in medullary thyroid carcinoma: a case report. *J. Nucl. Med.*, **31**, 1854–1855.


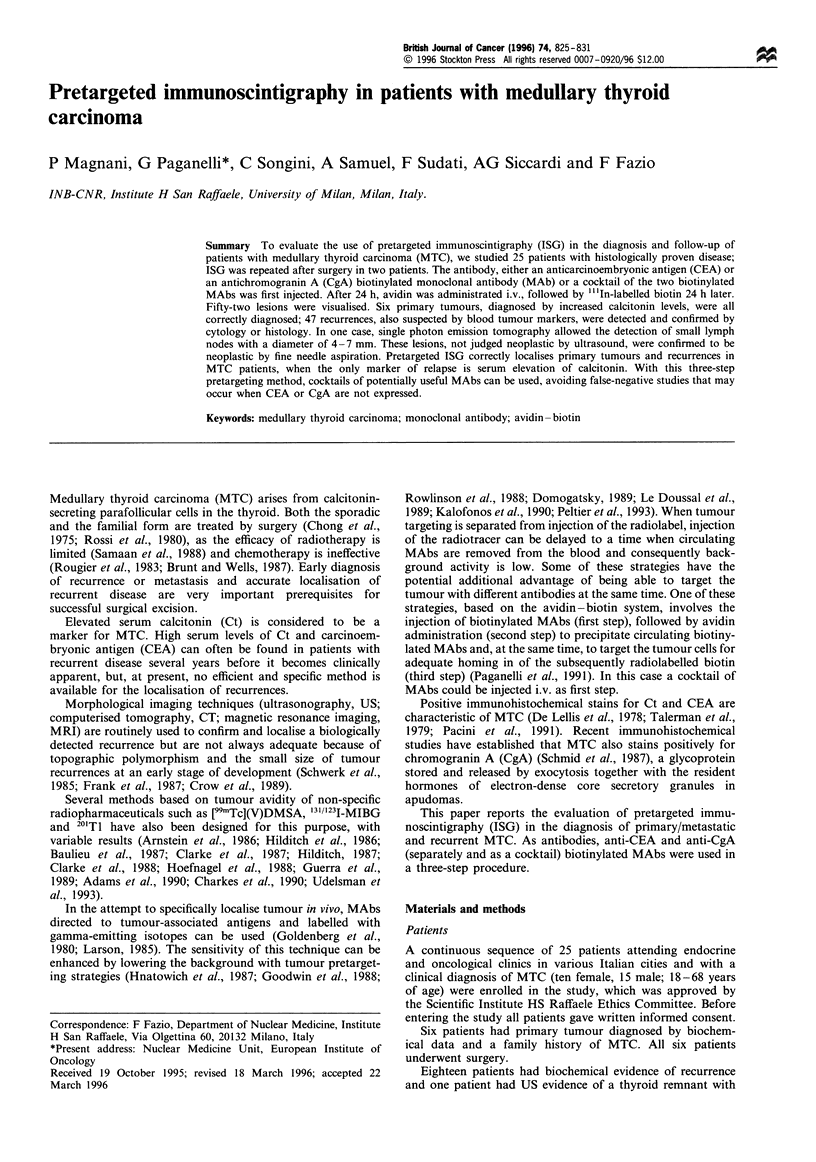

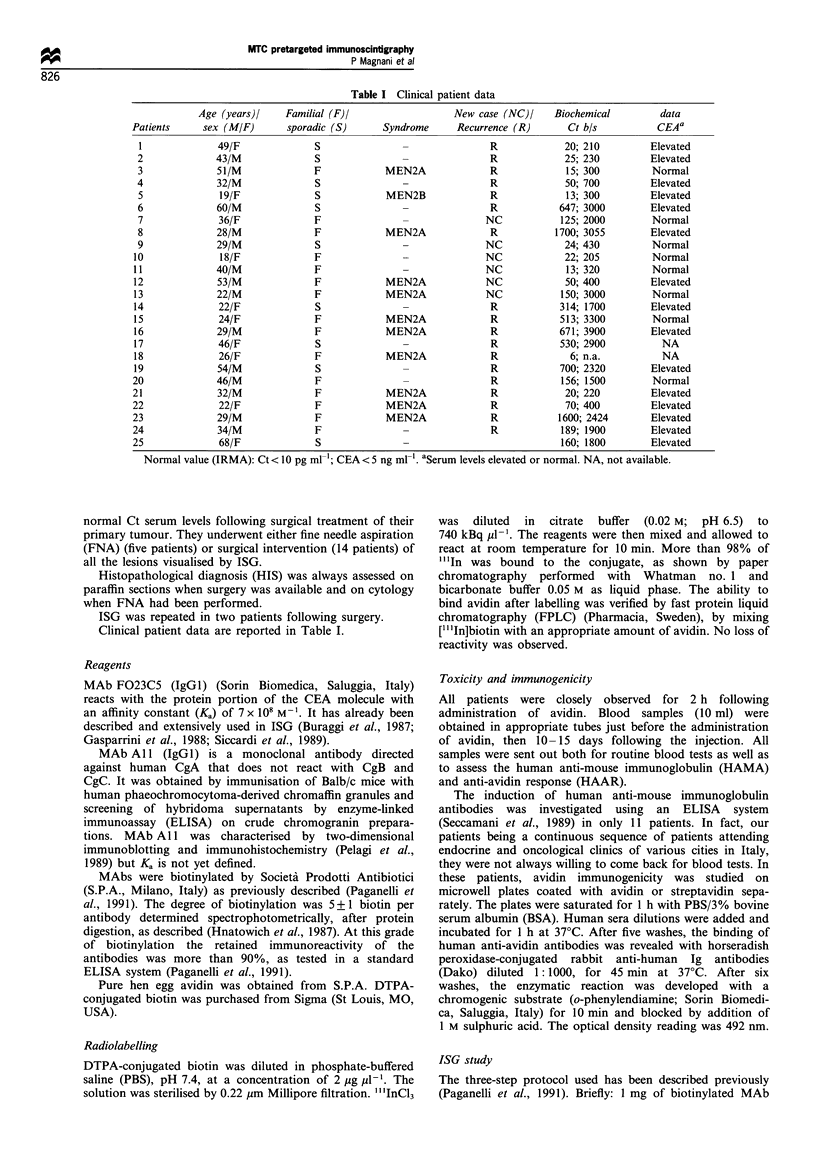

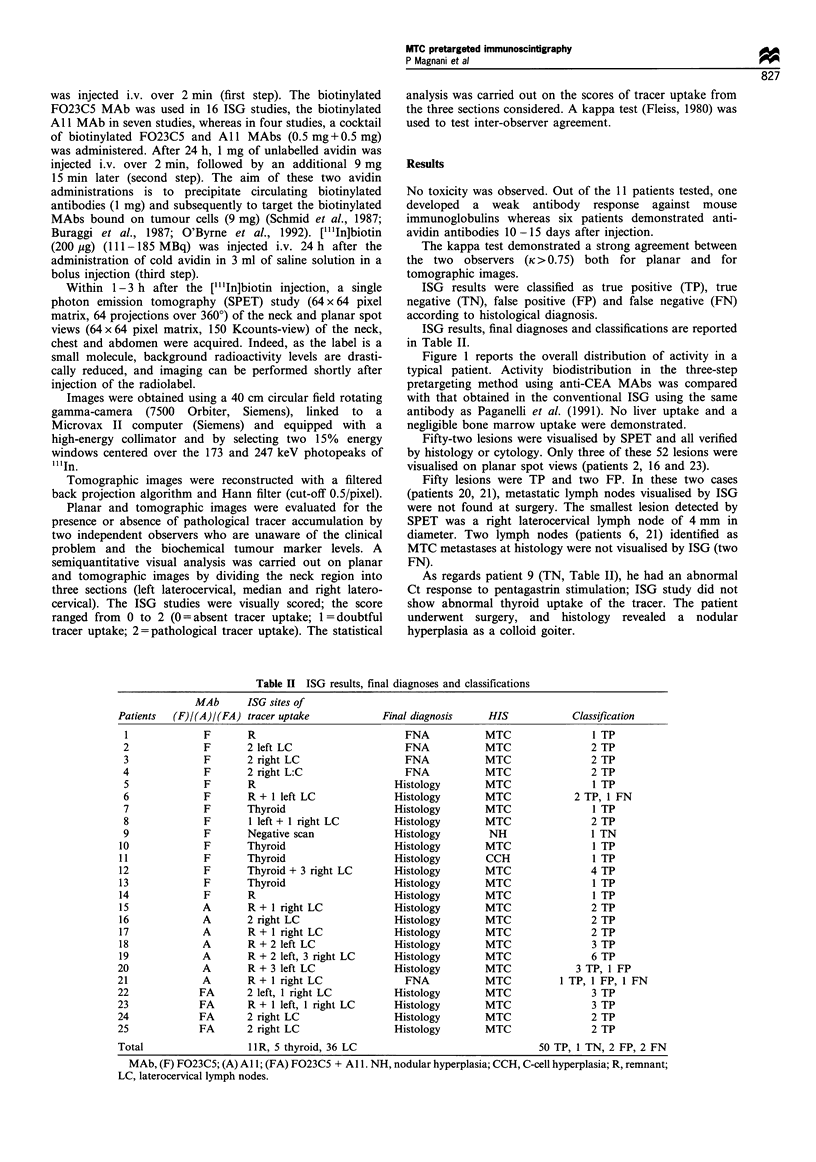

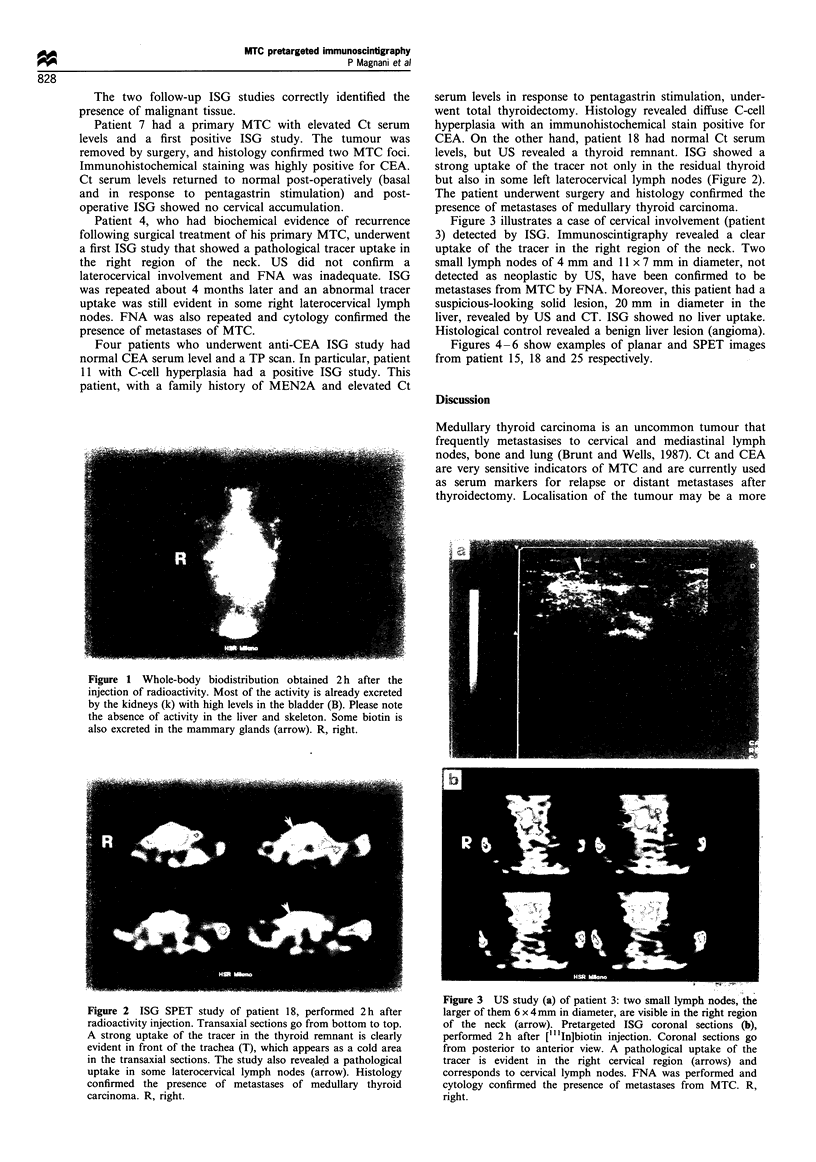

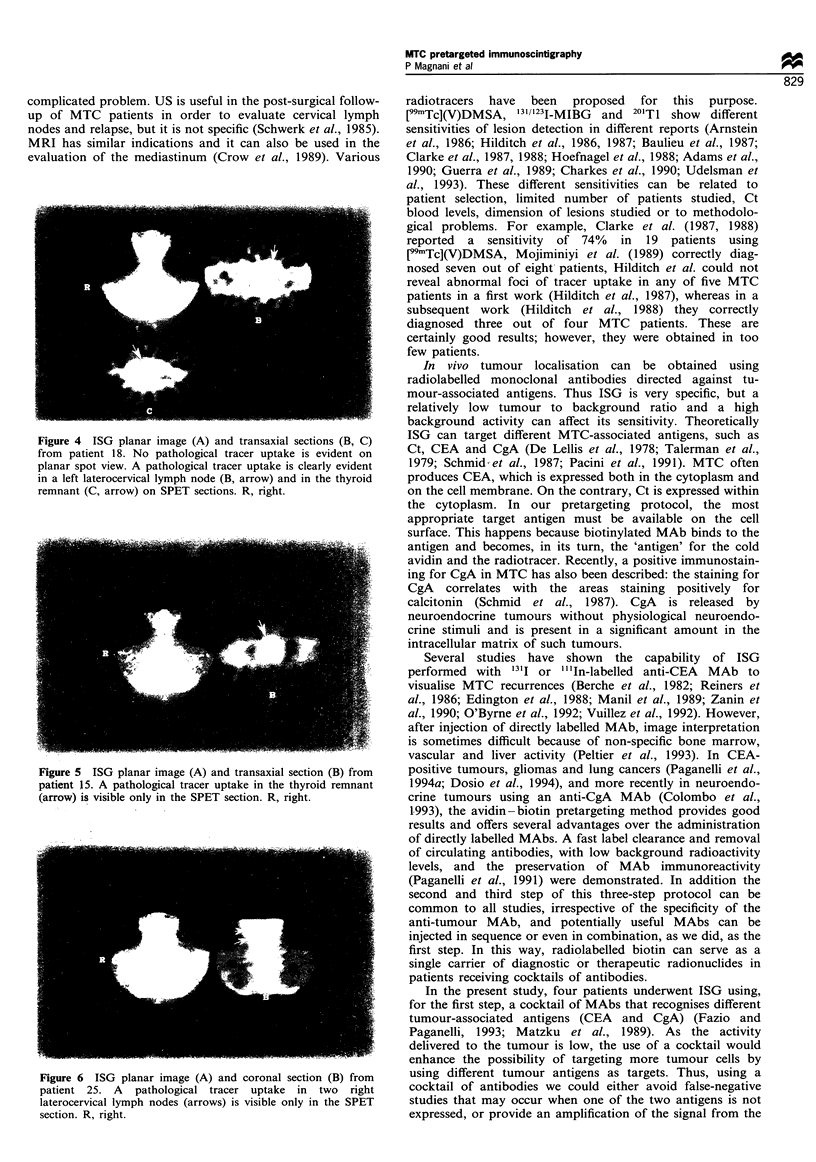

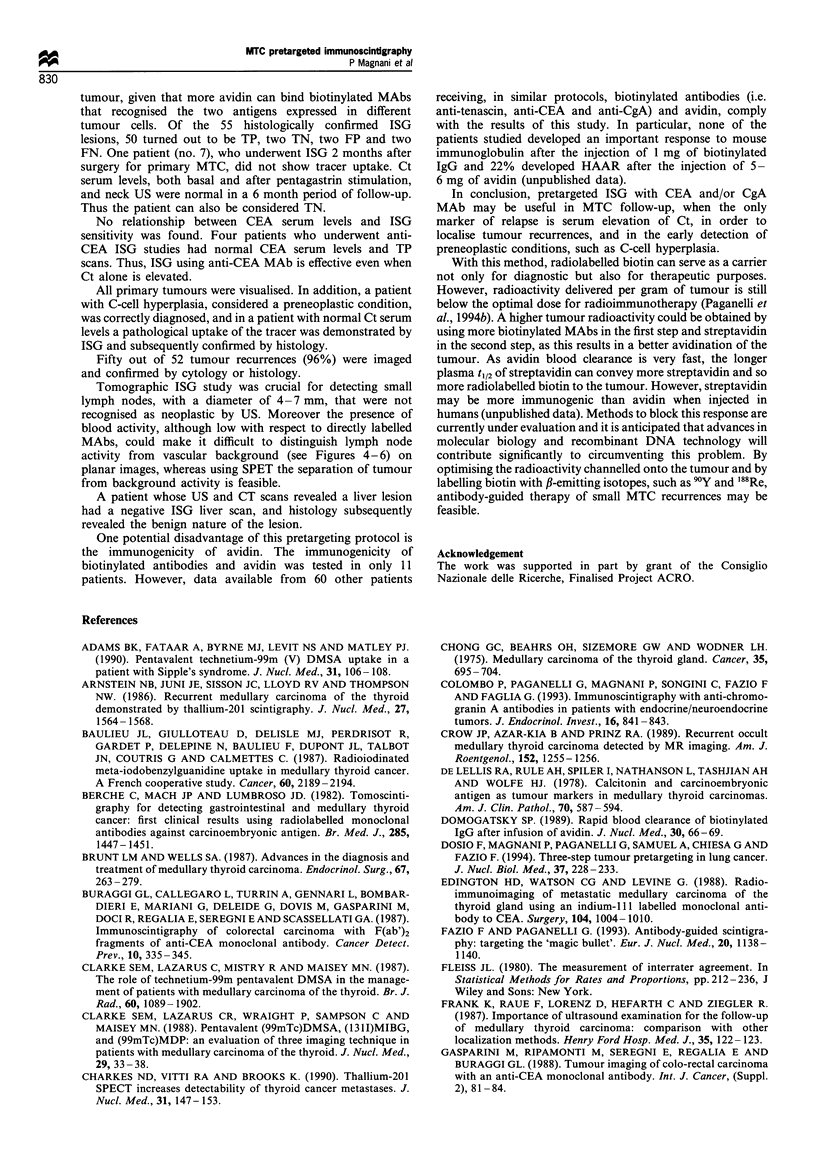

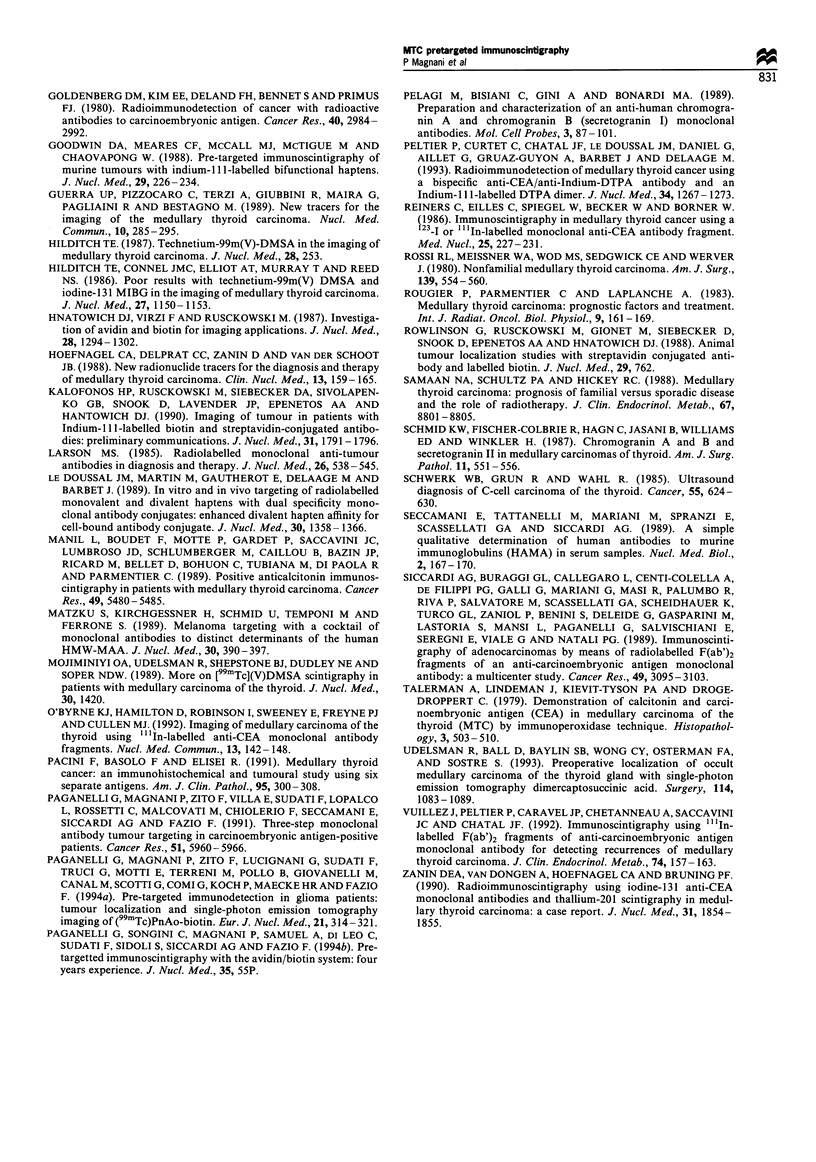


## References

[OCR_00837] Adams B. K., Fataar A., Byrne M. J., Levitt N. S., Matley P. J. (1990). Pentavalent technetium-99m (V)-DMSA uptake in a pheochromocytoma in a patient with Sipple's syndrome.. J Nucl Med.

[OCR_00843] Arnstein N. B., Juni J. E., Sisson J. C., Lloyd R. V., Thompson N. W. (1986). Recurrent medullary carcinoma of the thyroid demonstrated by thallium-201 scintigraphy.. J Nucl Med.

[OCR_00846] Baulieu J. L., Guilloteau D., Delisle M. J., Perdrisot R., Gardet P., Delépine N., Baulieu F., Dupont J. L., Talbot J. N., Coutris G. (1987). Radioiodinated meta-iodobenzylguanidine uptake in medullary thyroid cancer. A French cooperative study.. Cancer.

[OCR_00855] Berche C., Mach J. P., Lumbroso J. D., Langlais C., Aubry F., Buchegger F., Carrel S., Rougier P., Parmentier C., Tubiana M. (1982). Tomoscintigraphy for detecting gastrointestinal and medullary thyroid cancers: first clinical results using radiolabelled monoclonal antibodies against carcinoembryonic antigen.. Br Med J (Clin Res Ed).

[OCR_00862] Brunt L. M., Wells S. A. (1987). Advances in the diagnosis and treatment of medullary thyroid carcinoma.. Surg Clin North Am.

[OCR_00865] Buraggi G., Callegaro L., Turrin A., Gennari L., Bombardieri E., Mariani G., Deleide G., Dovis M., Gasparini M., Doci R. (1987). Immunoscintigraphy of colorectal carcinoma with F (ab')2 fragments of anti-CEA monoclonal antibody.. Cancer Detect Prev.

[OCR_00886] Charkes N. D., Vitti R. A., Brooks K. (1990). Thallium-201 SPECT increases detectability of thyroid cancer metastases.. J Nucl Med.

[OCR_00891] Chong G. C., Beahrs O. H., Sizemore G. W., Woolner L. H. (1975). Medullary carcinoma of the thyroid gland.. Cancer.

[OCR_00882] Clarke S. E., Lazarus C. R., Wraight P., Sampson C., Maisey M. N. (1988). Pentavalent [99mTc]DMSA, [131I]MIBG, and [99mTc]MDP--an evaluation of three imaging techniques in patients with medullary carcinoma of the thyroid.. J Nucl Med.

[OCR_00875] Clarke S. E., Lazarus C., Mistry R., Maisey M. N. (1987). The role of technetium-99m pentavalent DMSA in the management of patients with medullary carcinoma of the thyroid.. Br J Radiol.

[OCR_00899] Colombo P., Paganelli G., Magnani P., Songini C., Fazio F., Faglia G. (1993). Immunoscintigraphy with anti-chromogranin A antibodies in patients with endocrine/neuroendocrine tumors.. J Endocrinol Invest.

[OCR_00904] Crow J. P., Azar-Kia B., Prinz R. A. (1989). Recurrent occult medullary thyroid carcinoma detected by MR imaging.. AJR Am J Roentgenol.

[OCR_00910] DeLellis R. A., Rule A. H., Spiler I., Nathanson L., Tashjian A. H., Wolfe H. J. (1978). Calcitonin and carcinoembryonic antigen as tumor markers in medullary thyroid carcinoma.. Am J Clin Pathol.

[OCR_00917] Dosio F., Magnani P., Paganelli G., Samuel A., Chiesa G., Fazio F. (1993). Three-step tumor pre-targeting in lung cancer immunoscintigraphy.. J Nucl Biol Med.

[OCR_00922] Edington H. D., Watson C. G., Levine G., Tauxe W. N., Yousem S. A., Unger M., Kowal C. D. (1988). Radioimmunoimaging of metastatic medullary carcinoma of the thyroid gland using an indium-111-labeled monoclonal antibody to CEA.. Surgery.

[OCR_00928] Fazio F., Paganelli G. (1993). Antibody-guided scintigraphy: targeting of the "magic bullet".. Eur J Nucl Med.

[OCR_00938] Frank K., Raue F., Lorenz D., Herfarth C., Ziegler R. (1987). Importance of ultrasound examination for the follow-up of medullary thyroid carcinoma: comparison with other localization methods.. Henry Ford Hosp Med J.

[OCR_00946] Gasparini M., Ripamonti M., Seregni E., Regalia E., Buraggi G. L. (1988). Tumor imaging of colo-rectal carcinoma with an anti-CEA monoclonal antibody.. Int J Cancer Suppl.

[OCR_00958] Goldenberg D. M., Kim E. E., DeLand F. H., Bennett S., Primus F. J. (1980). Radioimmunodetection of cancer with radioactive antibodies to carcinoembryonic antigen.. Cancer Res.

[OCR_00962] Goodwin D. A., Meares C. F., McCall M. J., McTigue M., Chaovapong W. (1988). Pre-targeted immunoscintigraphy of murine tumors with indium-111-labeled bifunctional haptens.. J Nucl Med.

[OCR_00968] Guerra U. P., Pizzocaro C., Terzi A., Giubbini R., Maira G., Pagliaini R., Bestagno M. (1989). New tracers for the imaging of the medullary thyroid carcinoma.. Nucl Med Commun.

[OCR_00976] Hilditch T. E., Connell J. M., Elliot A. T., Murray T., Reed N. S. (1986). Poor results with technetium-99m (V) DMS and iodine-131 MIBG in the imaging of medullary thyroid carcinoma.. J Nucl Med.

[OCR_00986] Hnatowich D. J., Virzi F., Rusckowski M. (1987). Investigations of avidin and biotin for imaging applications.. J Nucl Med.

[OCR_00991] Hoefnagel C. A., Delprat C. C., Zanin D., van der Schoot J. B. (1988). New radionuclide tracers for the diagnosis and therapy of medullary thyroid carcinoma.. Clin Nucl Med.

[OCR_00993] Kalofonos H. P., Rusckowski M., Siebecker D. A., Sivolapenko G. B., Snook D., Lavender J. P., Epenetos A. A., Hnatowich D. J. (1990). Imaging of tumor in patients with indium-111-labeled biotin and streptavidin-conjugated antibodies: preliminary communication.. J Nucl Med.

[OCR_01001] Larson S. M. (1985). Radiolabeled monoclonal anti-tumor antibodies in diagnosis and therapy.. J Nucl Med.

[OCR_01005] Le Doussal J. M., Martin M., Gautherot E., Delaage M., Barbet J. (1989). In vitro and in vivo targeting of radiolabeled monovalent and divalent haptens with dual specificity monoclonal antibody conjugates: enhanced divalent hapten affinity for cell-bound antibody conjugate.. J Nucl Med.

[OCR_01009] Manil L., Boudet F., Motte P., Gardet P., Saccavini J. C., Lumbroso J. D., Schlumberger M., Caillou B., Bazin J. P., Ricard M. (1989). Positive anticalcitonin immunoscintigraphy in patients with medullary thyroid carcinoma.. Cancer Res.

[OCR_01019] Matzku S., Kirchgessner H., Schmid U., Temponi M., Ferrone S. (1989). Melanoma targeting with a cocktail of monoclonal antibodies to distinct determinants of the human HMW-MAA.. J Nucl Med.

[OCR_01026] Mojiminiyi O. A., Udelsman R., Shepstone B. J., Dudley N. E., Soper N. D. (1989). More on [99mTc](V)DMSA scintigraphy in patients with medullary carcinoma of the thyroid.. J Nucl Med.

[OCR_01031] O'Byrne K. J., Hamilton D., Robinson I., Sweeney E., Freyne P. J., Cullen M. J. (1992). Imaging of medullary carcinoma of the thyroid using 111In-labelled anti-CEA monoclonal antibody fragments.. Nucl Med Commun.

[OCR_01037] Pacini F., Basolo F., Elisei R., Fugazzola L., Cola A., Pinchera A. (1991). Medullary thyroid cancer. An immunohistochemical and humoral study using six separate antigens.. Am J Clin Pathol.

[OCR_01050] Paganelli G., Magnani P., Zito F., Lucignani G., Sudati F., Truci G., Motti E., Terreni M., Pollo B., Giovanelli M. (1994). Pre-targeted immunodetection in glioma patients: tumour localization and single-photon emission tomography imaging of [99mTc]PnAO-biotin.. Eur J Nucl Med.

[OCR_01040] Paganelli G., Magnani P., Zito F., Villa E., Sudati F., Lopalco L., Rossetti C., Malcovati M., Chiolerio F., Seccamani E. (1991). Three-step monoclonal antibody tumor targeting in carcinoembryonic antigen-positive patients.. Cancer Res.

[OCR_01060] Pelagi M., Bisiani C., Gini A., Bonardi M. A., Rosa P., Marè P., Viale G., Grazia Cozzi M., Salvadore M., Zanini A. (1989). Preparation and characterization of anti-human chromogranin A and chromogranin B (secretogranin I) monoclonal antibodies.. Mol Cell Probes.

[OCR_01068] Peltier P., Curtet C., Chatal J. F., Le Doussal J. M., Daniel G., Aillet G., Gruaz-Guyon A., Barbet J., Delaage M. (1993). Radioimmunodetection of medullary thyroid cancer using a bispecific anti-CEA/anti-indium-DTPA antibody and an indium-111-labeled DTPA dimer.. J Nucl Med.

[OCR_01074] Reiners C., Eilles C., Spiegel W., Becker W., Börner W. (1986). Immunoscintigraphy in medullary thyroid cancer using an 123I- or 111In-labelled monoclonal anti-CEA antibody fragment.. Nuklearmedizin.

[OCR_01079] Rossi R. L., Cady B., Meissner W. A., Wool M. S., Sedgwick C. E., Werber J. (1980). Nonfamilial medullary thyroid carcinoma.. Am J Surg.

[OCR_01086] Rougier P., Parmentier C., Laplanche A., Lefevre M., Travagli J. P., Caillou B., Schlumberger M., Lacour J., Tubiana M. (1983). Medullary thyroid carcinoma: prognostic factors and treatment.. Int J Radiat Oncol Biol Phys.

[OCR_01103] Schmid K. W., Fischer-Colbrie R., Hagn C., Jasani B., Williams E. D., Winkler H. (1987). Chromogranin A and B and secretogranin II in medullary carcinomas of the thyroid.. Am J Surg Pathol.

[OCR_01109] Schwerk W. B., Grün R., Wahl R. (1985). Ultrasound diagnosis of C-cell carcinoma of the thyroid.. Cancer.

[OCR_01114] Seccamani E., Tattanelli M., Mariani M., Spranzi E., Scassellati G. A., Siccardi A. G. (1989). A simple qualitative determination of human antibodies to murine immunoglobulins (HAMA) in serum samples.. Int J Rad Appl Instrum B.

[OCR_01121] Siccardi A. G., Buraggi G. L., Callegaro L., Colella A. C., De Filippi P. G., Galli G., Mariani G., Masi R., Palumbo R., Riva P. (1989). Immunoscintigraphy of adenocarcinomas by means of radiolabeled F(ab')2 fragments of an anti-carcinoembryonic antigen monoclonal antibody: a multicenter study.. Cancer Res.

[OCR_00915] Sinitsyn V. V., Mamontova A. G., Checkneva Y. Y., Shnyra A. A., Domogatsky S. P. (1989). Rapid blood clearance of biotinylated IgG after infusion of avidin.. J Nucl Med.

[OCR_01130] Talerman A., Lindeman J., Kievit-Tyson P. A., Dröge-Droppert C. (1979). Demonstration of calcitonin and carcinoembryonic antigen (CEA) in medullary carcinoma of the thyroid (MCT) by immunoperoxidase technique.. Histopathology.

[OCR_01137] Udelsman R., Ball D., Baylin S. B., Wong C. Y., Osterman F. A., Sostre S. (1993). Preoperative localization of occult medullary carcinoma of the thyroid gland with single-photon emission tomography dimercaptosuccinic acid.. Surgery.

[OCR_01144] Vuillez J. P., Peltier P., Caravel J. P., Chetanneau A., Saccavini J. C., Chatal J. F. (1992). Immunoscintigraphy using 111In-labeled F(ab')2 fragments of anticarcinoembryonic antigen monoclonal antibody for detecting recurrences of medullary thyroid carcinoma.. J Clin Endocrinol Metab.

[OCR_01151] Zanin D. E., van Dongen A., Hoefnagel C. A., Bruning P. F. (1990). Radioimmunoscintigraphy using iodine-131-anti-CEA monoclonal antibodies and thallium-201 scintigraphy in medullary thyroid carcinoma: a case report.. J Nucl Med.

